# Recent upgrades of the harmonic radar for the tracking of the Asian yellow‐legged hornet

**DOI:** 10.1002/ece3.3053

**Published:** 2017-05-18

**Authors:** Daniele Milanesio, Maurice Saccani, Riccardo Maggiora, Daniela Laurino, Marco Porporato

**Affiliations:** ^1^ Politecnico di TorinoDipartimento di Elettronica e Telecomunicazioni (DET)TorinoItaly; ^2^ Dipartimento di Scienze Agrarie, Forestali e Alimentari (DISAFA)Universitá degli Studi di TorinoTorinoItaly

**Keywords:** Asian hornet, harmonic radar, insect tracking, *Vespa velutina*

## Abstract

The yellow‐legged Asian hornet is an invasive species of wasps, indigenous of the South‐East Asia but quickly spreading in Southern Europe. Because of its exponential diffusion and its serious threat to the local honey bee colonies and to humans as well, restraint measures are under investigation. Among them, the harmonic radar described in (Ecology and Evolution, 6, 2016 and 2170) already proved to be a quite effective way to follow the hornets to their nests; it is in fact capable of tracking the flying trajectory of these insects, once equipped with a small transponder, in their natural environment. The aforementioned harmonic radar was upgraded after a period of intense experimentation; the capture of the hornets was enhanced as well, and other improvements were adopted in the mounting procedure of the transponder. Thanks to these upgrades, the flying capabilities of the hornets were not reduced and a huge collection of data was recorded. The main upgrade to the radar was the adoption of the vertical polarization of the radiated field, with the consequent redesign and manufacturing of the antennas and the different mounting of the transceiver on the insect. The installation of the radar on a telescopic tower drastically improved the maneuverability of the system and the capability to follow the insects’ preferential flying directions. Eventually, the system was able to produce much more continuous traces with a clear indication of the most probable position of the nest. The maximum range of detection was also increased to 150 m.

## 1 INTRODUCTION

*Vespa velutina* arrived in Europe in 2004 (Haxaire, Bouguet, & Tamisier, 2006) and quickly spread despite numerous attempts to control it (Demichelis, Manino, Minuto, Mariotti, & Porporato, 2014; Rome et al., 2013); different types of traps and baits were used to restrain it, together with the destruction of the found colonies (see Monceau, Bonnard, & Thiéry, 2014). Recently, the yellow‐legged Asian hornet has been observed also in new countries such as England and Germany.

As documented in Bertolino, Lioy, Laurino, Manino, and Porporato (2016), the area occupied by the these hornets in Italy increased from 205 km^2^ in 2013 to 930 km^2^ in 2015; the front line was at 55 km along the coast from the French border in 2015, with a linear spread of 18.3 ± 3.3 km/year. In few cases, the dispersion of the invasive insect was clearly human‐mediated. A cluster analysis of the range allowed the identification of 17 core areas used by the hornets, with a mean nest density of about 2.9–3.5 nests/km^2^. These numbers are of paramount importance not only to monitor the evolution of the diffusion of the yellow‐legged hornet in Italy but also to establish an early warning and rapid response system and, therefore, to set up an effective management plan.

Robinet, Suppo, and Darrouzet (2017) showed that increasing the percentage of destroyed nests from 30% to 60% could reduce the species spread by 17% and its nest density by 29%. If 95% of nests were to be destroyed, the species spread and nest density could decline by 43% and 53%, respectively.

These encouraging data, together with the observation of limited effectiveness of the control activities so far implemented, keep enforcing the idea that the development of a radar system (Milanesio, Saccani, Maggiora, Laurino, & Porporato, 2016) to track the flight of the hornets and find the nests could be extremely useful.

## 2 MATERIALS AND METHODS

While referring the interested reader to Milanesio et al. (2016) for a detailed analysis of the first version of this entomological harmonic radar, we would like to herein recall the basic principles.

An harmonic radar launches a wave at a fixed frequency and usually receives the second harmonic of the reflected signal, produced by a nonlinear device (referred as tag or transponder) mounted on the target. This technique allows to discriminate the target from all other elements present in the environment under test, for they basically reflect the impinging wave at the same harmonic.

As also alluded in Milanesio et al. (2016), this radar operates in the quite challenging hilly and woody environment of the Western Liguria, Italy, in a far more complex condition than the one described in the pioneering work of Riley and Smith (2002). This difference practically translates in the need of less directive antennas in the vertical plane, that is, capable of covering a larger angle in elevation with the disadvantage of a reduced gain.

Finally, the radar detections should be analyzed in a statistical way; a relevant number of tagged insects should be taken into account to provide preferential directions of flight along which the radar itself can be moved and get closer to the hornets’ nests.

**Figure 1:**
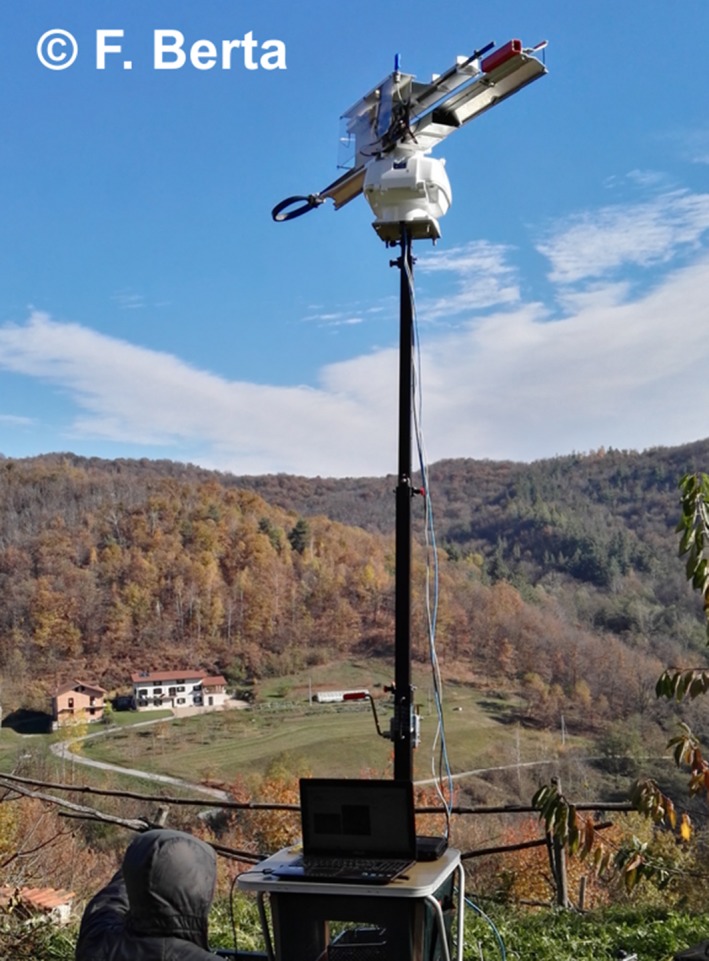
New setup of the harmonic radar for the yellow‐legged hornet tracking; the radar currently operates on top of a telescopic portable tower which allows to get rid of obstacles shorter than about 6 m

**Figure 2:**
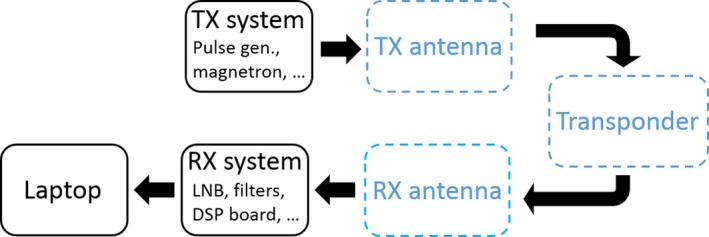
Block diagram of the overall harmonic radar: The blue dashed blocks represent the portions of the system which have been upgraded with respect to the previous version of the radar. For sake of simplicity, all the steps present in the transmitting and receiving chain (with the exception of the antennas) have been grouped into a single block

Figure 1 depicts the new radar configuration: The reader should immediately notice that a telescopic tower (up to 6 m of maximum vertical extension) was successfully inserted to physically get rid of closest obstructions. As for the previous version, a 12‐V car battery, powering all electronics, and a laptop, performing the real‐time analysis of the received signals, complete the system. Figure 2 documents the upgrades from a functional point of view: The blue dashed blocks represent the portions of the system which have been upgraded with respect to previous implementation of the entomological radar.

### 2.1 Upgrades of the TX system

The transmission chain still relies on a commercial off‐the‐shelf marine radar built by FURUNO (www.furuno.com), which allowed to lower the costs of the system development.

A new module was added right before the TX antenna, namely a commercial WR90 waveguide filter, with the goal of reducing the unwanted emissions at 18.82 GHz. To be more specific, even though the TX antenna is supposed to emit only at 9.41 GHz, there was a small emission at the second harmonic, that is, 18.82 GHz; the echoes from the environment of this 18.82‐GHz emission directly disturbed the received signal. The introduction of the filter helped to considerably reduce this source of clutter. The working frequency of the filter is between 8.2 and 12.4 GHz, with an insertion loss of 0.2 dB at 9.41 GHz and of 60 dB at 18.82 GHz.

The main upgrade of the TX system consisted in the adoption of a new antenna with vertical polarization instead of the previous horizontal one. This change was driven by the fact that, if one assumes that the tag can be successfully mounted on the back of the hornet in vertical direction (as done for instance in Riley and Smith (2002) and herein described in the correspondent subsection), it is definitely more convenient than the horizontal polarization; in fact, as long as the tag stays vertical, that is, basically most of the time during the flight of the insect, the relative horizontal attitude of the specimen with respect to the radar is practically irrelevant, the tag will be detected in any case with negligible polarization loss.

**Figure 3:**
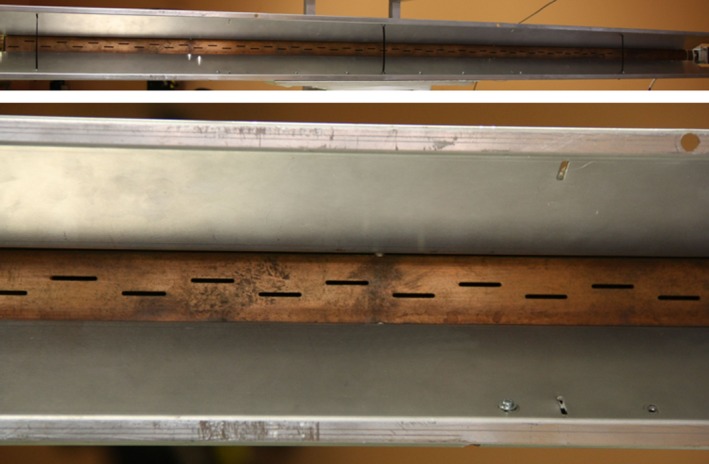
New TX vertically polarized slotted waveguide antenna (top) with a detail of the slots (bottom) carved on its surface. The position of the slots with respect to the center of the waveguide and their length allow to choose the amount of power delivered by each radiating element and, eventually, to optimize the radiated field on the azimuthal plane

**Figure 4:**
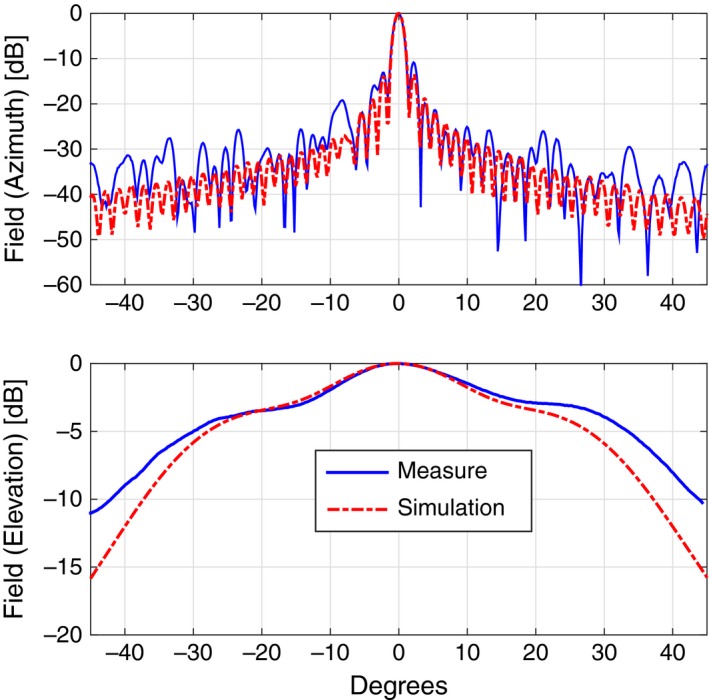
Comparison of the radiated fields in azimuth (top) and elevation (bottom) between the CST‐MWS simulated design (dashed red curve) and the measured data (plain blue curve) for the TX antenna

Coming to the details, the new TX antenna, a 150‐cm‐long slotted waveguide with 50 radiating elements (slots), was designed with the help of the commercial code CST‐MWS (www.cst.com) and entirely manufactured by the Antenna and Electromagnetic Compatibility Laboratory (LACE) of the Department of Electronics of Politecnico di Torino. Figure 3 shows the final geometry (top) with a zoomed view of few of the radiating elements (bottom), uniformly tapered along the waveguide direction to reduce secondary lobes. Figure 4 reports the simulated and measured radiation diagram, which roughly corresponds to the one produced by the previous horizontally polarized FURUNO antenna; the gain on the direction of main radiation is about 26.5 dB, 2 dB less than the FURUNO antenna, mainly due to the reduced number of radiating elements, that is, 50 versus 72. The reader should also notice from Figure 3 that the new antenna still mounts the flanges already present in the previous launcher, in order to implement a vertical sectoral horn and, therefore, to get a more directive field on the vertical plane (main lobe in elevation of about 30° at −3 dB). A summary of the antenna performances is listed in Table 1.

**Table 1:** Features of transmitting and receiving antennas mounted on the upgraded radar

TX antenna polarization	Vertical
TX antenna hor. half power beam width (HPBW)	1.4°
TX antenna vert. half power beam width	24°
TX antenna gain	26.6 dBi
RX antenna polarization	Vertical
RX antenna hor. half power beam width (HPBW)	1.5°
RX antenna vert. half power beam width	19.6°
RX antenna gain	27.3 dBi

**Figure 5:**
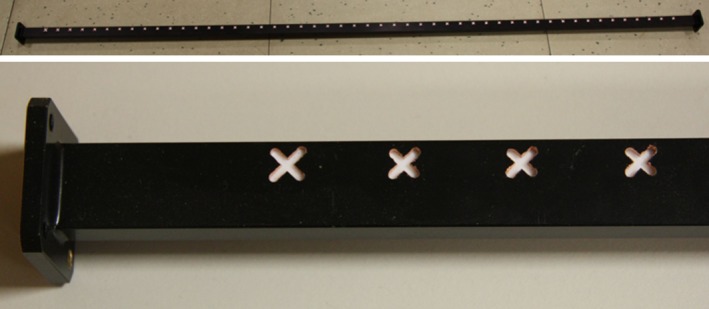
Circularly polarized slotted waveguide antenna (top) with a detail of the slots (bottom) carved on its surface. The position and size of the slots allow to optimize the radiated field on the azimuthal plane, while the different length of the two arms of the cross is responsible for the produced polarization

**Figure 6:**
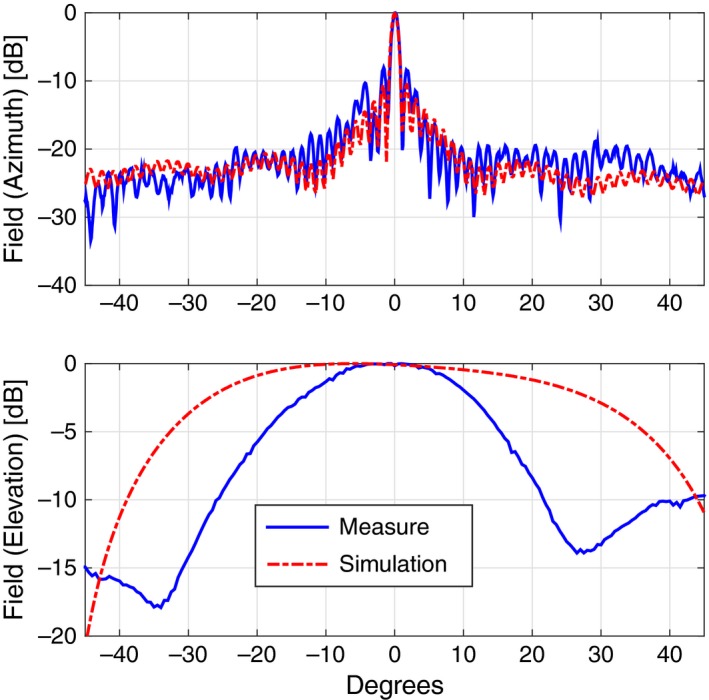
Comparison of the radiated fields in azimuth (top) and elevation (bottom) between the CST‐MWS simulated design (dashed red curve) and the measured data (plain blue curve)

Before selecting the vertically polarized antenna, an intermediate step with launchers with circular polarization was tested as well. Circular polarization of the emitted wave removes all sorts of limitations about the relative orientation of the tag with respect to the radar. However, no flanges can be used to increase the antenna directivity (circular polarization will be lost) and the manufactured launcher, even though showing the expected properties in terms of polarization, severely lacked in terms of gain (21 dB along the direction of maximum radiation). The rather consistent reduction of the TX antenna gain (together with the about same reduction coming from the RX antenna) determined a consequent decrease in the maximum distance of detection, going down to about 70 m from the radar (approximately half of the distance achieved with linearly polarized antennas). Despite being discarded so far (it may come in hand if one can compensate for the distance in other ways or it is not interested in huge distances at all), this antenna is quite unique in literature and deserves to be briefly presented. As documented in Figure 5, the 150‐cm‐long antenna is once more a slotted waveguide, a WR75 to be precise, but it is filled with Teflon (dielectric constant equal to 2.1) in order to guarantee the correct distance at 9.41 GHz, in guided wavelengths, between the radiating elements. 50 cross‐slots are carved on the upper half of the broad side of the waveguide, providing both the required circular polarization by optimizing the length of the two arms, and acceptable side lobes level by changing their displacement with respect to the center of the waveguide itself. To conclude, Figure 6 reports the radiated field; a not negligible difference in the main lobe amplitude was noted in the elevation plane and it is currently under investigation.

All other details about the TX system are not changed and can be found in Milanesio et al. (2016). In particular, we remind that the pulse repetition frequency of the radar is 3 kHz, the peak output power is 25 kW, the pulse width is 100 ns, and the rotations per minute are 48.

### 2.2 Upgrades of the RX system

The RX system is conceptually made up of the same stages described in figure 5 of Milanesio et al. (2016), with a received signal accuracy of 1.5 m in range and 0.1° in angle, out of a raw radar resolution of 15 m. The radar receiver sensitivity depends on many factor, as explained in Milanesio et al. (2016), and can be estimated around −90 dBm. In short, the 18.82‐GHz received signal is first amplified and then mixed with the local oscillator of a low noise down‐converter block. Once demodulated and filtered, the signal is rectified by a logarithmic detector, digitized, and processed by a high‐performance digital signal processor board. Eventually, the output data are visualized in real time on a laptop. Despite the similarities in the flowing chart, new receiving antennas were tested and a huge work was done to optimize the transponder configuration and mounting procedure.

#### 2.2.1 The transponder

The adopted transponder is made up of two copper pieces connected to a zero‐bias Schottky diode that generates harmonics of the received signal, that is, from an incoming signal at 9.41 GHz to the strongest generated signal at 18.82 GHz. The best configuration was found with a 0.25‐mm‐diameter wire and with a total length of 16 mm, with symmetrical branches on both sides of the diode; the overall weight of the transponder is 12 mg. We refer the interested reader to Milanesio et al. (2016) for the analysis of the transponder performances.

**Figure 7:**
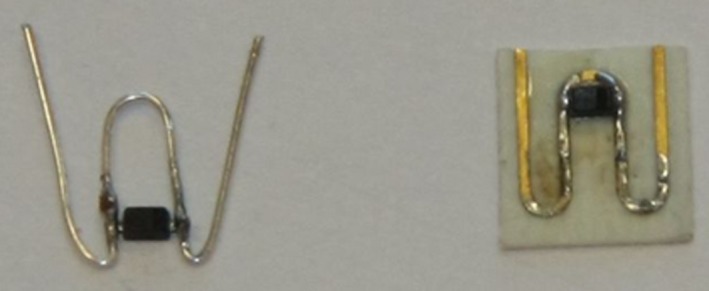
Two implementations of the “w‐shaped” transponder, manufactured with a bent copper wire (left) and with a printed wire (right)

As explained in Milanesio et al. (2016), the “loop” configuration cannot generate a retransmitted signal when the tag polarization is orthogonal to the radar antenna polarization, for instance, in case the transponder is installed horizontally, when the hornet is flying perfectly to or from the radar. This limitation was already removed by the “cross”‐implementation, which however has the disadvantage of the increased weight of the transponder. A third configuration, named “w‐shaped,” was studied in 2016 in order to get rid of the same problem with less drawbacks. Figure 7 documents two versions of the “w‐shaped” tag, one realized by simply bending the copper wire (on the left) and the second obtained with a printed wire on a dielectric substrate (on the right). In both cases, the shape of the wire allowed the tag to be detected in any orientation, clearly as long as its plane stayed horizontal. The first implementation appeared to be unstable; that is, it was quite difficult to manufacture two tags with the same properties; some of them were omnidirectional on the horizontal plane and others were almost not visible in some orientations. On the contrary, the printed approach permitted the perfect reproducibility of the tag and its properties but not all the hornets equipped with it could fly, due to its dimension interfering with the insect wings. An additional drawback is that printed tags are sensitive to the environment (see US patent 6456228B1, chapter “Disclosure of invention,” www.google.com/patents/US6456228); to me more specific, their respective impedances are influenced by the surroundings of the antenna resulting in a degraded conversion efficiency.

**Figure 8:**
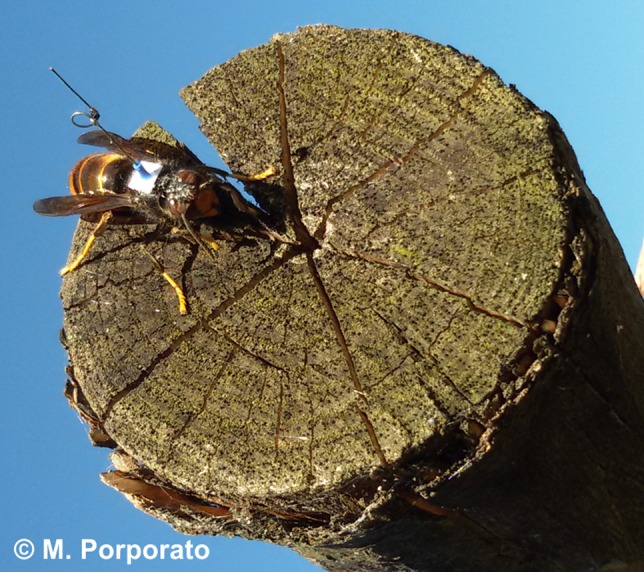
“Loop” tag mounted in vertical position on a hornet. To simplify the mounting procedure, the tag was previously glued to a small paper disk and held firm with the help of the external copper wire rubber covering

Given the persisting issues, as anticipated in Milanesio et al. (2016), we eventually decided to move to a vertical “loop” tag (as in Riley & Smith, 2002); Figure 8 shows the tag mounted on a hornet. To ease the mounting procedure, the tag was also preemptively disposed on a rigid base of approximately 4 mm^2^. This setup worked very well, providing several detections per flight and, in general, far better results than the previous configuration based on horizontally polarized antennas and tags.

**Figure 9:**
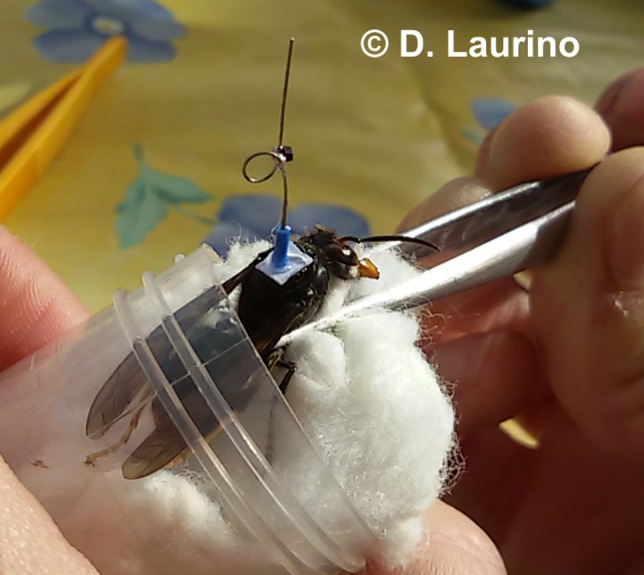
Mounting procedure: Once captured, the hornets are put in a Falcon tube, immobilized with a cotton swab and a pair of tweezers, and loaded with the tag, previously mounted on a small paper support with a rubber cylindrical cladding

In addition to the tests on the shape of the tag, we also optimized the mounting procedure. We started with a strong commercial glue which required about 20 s to create a reliable bond between the tag and the specimen; in order not to stress the insects during this period and to prevent them from moving, a small dose of carbon dioxide was necessary to slightly anesthetize them. The amount of glue resulted to be a rather delicate parameter: It had to be enough to prevent the hornet from removing the tag with its legs, above all in the very first seconds, but not too much to become a burden and obstruct the flight. Furthermore, the anesthesia seemed to worsen the recovery of the hornets, which appeared to be stunned for few minutes after their release. We finally tested a product adopted in orthodontics, namely Transbond™ XT (3M Unitek, Italy), which sticks also on wet surfaces and polymerizes in few seconds using a polymerization lamp, with a high‐intensity blue light UV 1,600 mW/cm^2^. With this tool, it was possible to reliably mount the tag in less than 8 s without any sort of anesthesia (no need to slow down their metabolism keeping them at low temperature either); the hornets were captured in front of the hives, put in a Falcon tube, immobilized with a cotton swab and a pair of tweezers, and loaded with the tag. Figure 9 shows the aforementioned mounting setup. This procedure had no visible impact on the hornet, which started to fly immediately after its release.

#### 2.2.2 The RX antenna

**Figure 10:**
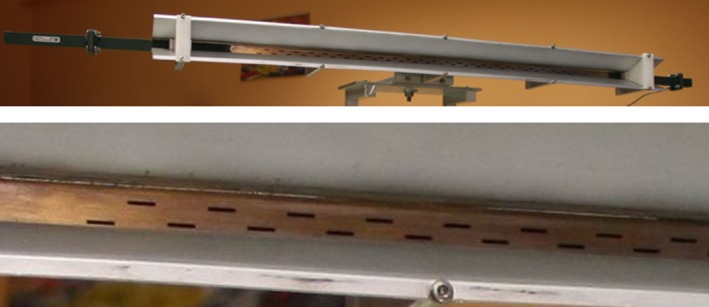
New RX vertically polarized slotted waveguide antenna (top) with a detail of the slots (bottom) carved on its surface

**Figure 11:**
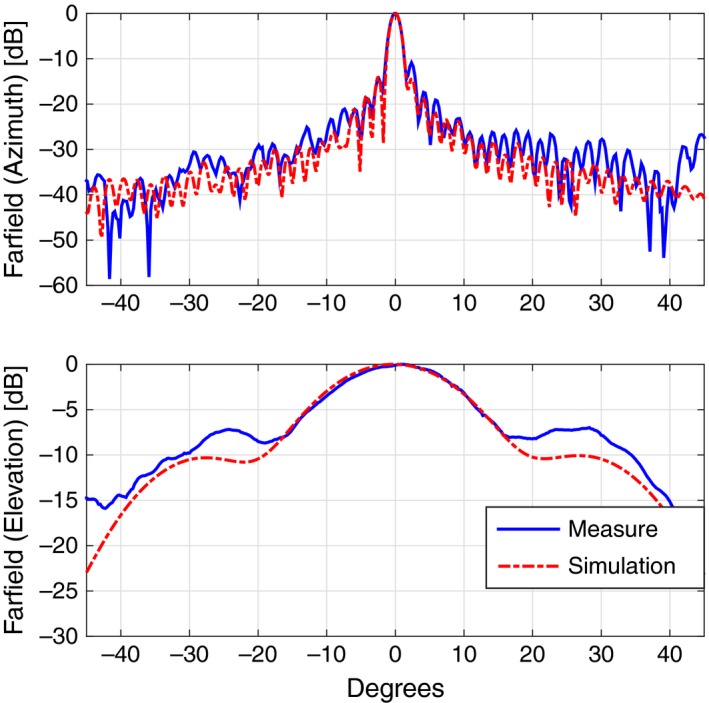
Comparison of the radiated fields in azimuth (top) and elevation (bottom) between the CST‐MWS simulated design (dashed red curve) and the measured data (plain blue curve) for the RX antenna

Similarly to the TX launcher, also for the receiving antenna at 18.82 GHz, we designed and manufactured a vertically polarized slotted waveguide. Figure 10 shows the 100‐cm‐long WR51 with its flanges, that is, a sectoral horn added to get a more directive field on the vertical plane. 50 slots are carved on the broad side of the waveguide for a total length of approximately 55 cm; as the reader can notice, the distance of the slots from the midplane of the waveguide varies along the direction of the launcher itself in order to have a uniform distribution of power. Figure 11 reports the comparison between the simulated geometry and the realized one in terms of radiated field, while Table 1 summarizes the main antenna parameters.

**Figure 12:**
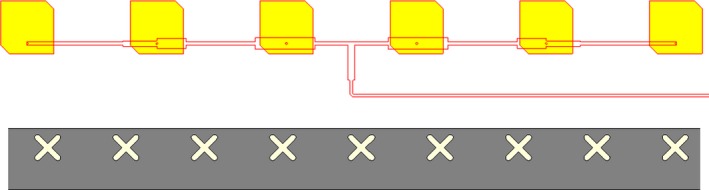
Zoomed view of the RX circularly polarized microstrip antenna (top) and of the slotted waveguide antenna (bottom). For sake of clarity, the beam forming network of the microstrip antenna is superimposed even though it is located in a different layer

**Figure 13:**
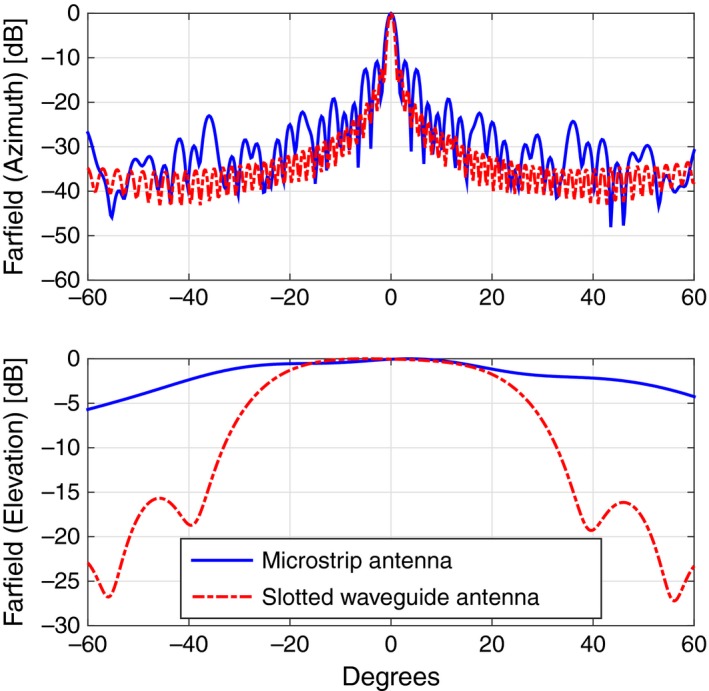
Comparison of the radiated fields in azimuth (top) and elevation (bottom) between the CST‐MWS simulated design of the circularly polarized microstrip antenna (plain blue curve) and the slotted waveguide antenna (dashed red curve)

We also designed two alternative solutions for the circularly polarized launcher: The first one was a microstrip antenna, while the second one was again a slotted waveguide, as documented in Figure 12. For the same reasons mentioned while treating the TX antenna, that is, too wide main lobe in elevation and therefore poor gain, also in this case we decided not to proceed on with this configuration; nevertheless, we would like to spend here few words describing their design, even at a theoretical stage. The microstrip antenna is made up of four copper layers, with two intermediate layers of dielectric substrate in between, namely Rogers RO4350 with dielectric constant equal to 3.66, and a central layer of IS400, a temperature‐resistant base material. The radiating element is a square patch, whose opposite corners are cut to produce the circular polarization; 48 patches are positioned on one side of the multilayer antenna and constitute the radiating part of it. On the other end of the stack, there is the beam forming network, that is, a set of 50 Ω lines and quarter wavelength adapters, properly adjusted to provide a uniform distribution of power and to set all radiating elements to the same phase. Patches and the beam forming network are then linked through metallic vias which cross all layers. The slotted microstrip antenna is built with a WR34 waveguide filled with Teflon ($\epsilon_{r}=2.1$), along which 50 cross‐slots are carved; a uniform distribution of power is again enforced by changing the size of the slots and their offset with respect to the midplane of the waveguide. As one can observe in Figure 13, while the radiated field in the azimuthal plane is quite good, the lobe in the elevation plane is definitely too wide, due to absence of the flanges; the antenna gain in the direction of maximum radiation is 21.6 dB for the microstrip antenna and 23.2 dB for the slotted waveguide, more than 3 dB less than the aforementioned vertically polarized launcher.

## 3 RESULTS

As in Milanesio et al. (2016), on‐field tests were performed nearby Dolceacqua, in the inland of the Ligurian coast, few kilometers from the France border, where the diffusion of the Asian hornet is nowadays quite intense. The area under analysis was about 300 m by 300 m, presented a not negligible slope of approximately 10% and had a discrete amount of tall trees and obstacles (poles, fences, houses, etc.).

**Figure 14:**
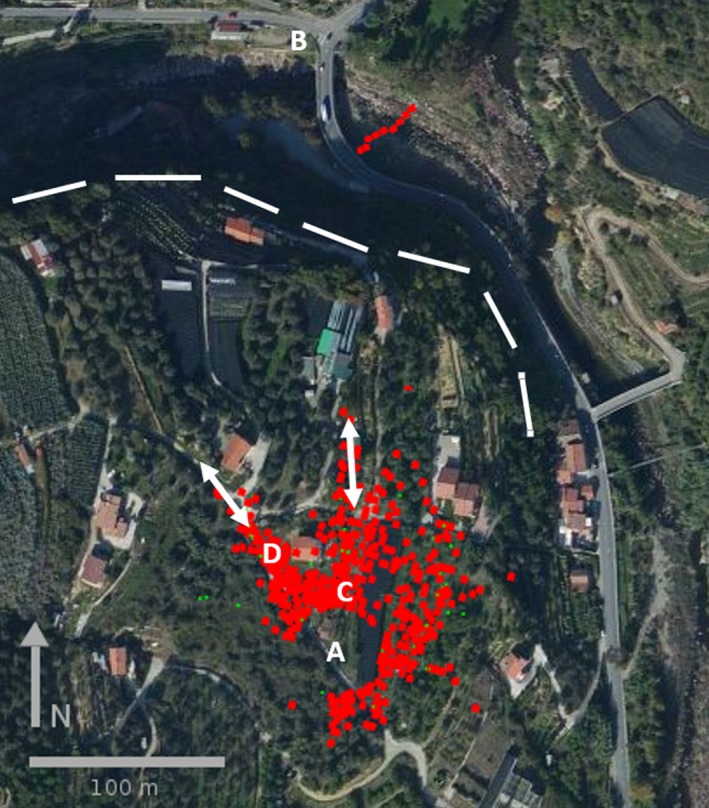
Examples of tracked insect flights during the on‐field campaign. Points “A” and “B” indicate the position of the radar during the two phases, point “C” is the position of the beehives, while “D” stands for a blooming medlar tree where several of the tagged hornets used to rest for a while and feed before flying away. The two arrows are the directions along which most of the insects were observed to reenter the detection range of the system, while the dashed line indicates the start of the steep slope of the hill

Several testing campaigns were performed throughout 2016 to access the upgrades described in the previous sections; we will report in this paper only the final acquisition from the 17th of November, where, despite the not too favorable weather conditions, we had the opportunity to test the final version of the system. In less than three hours around noon, we tagged almost 50 hornets and we recorded an astonishing amount of tracks, allowing us to describe the flight trajectories in such a complex environment. Figure 14 summarizes all the detections recorded during that campaign. The radar was first mounted in position “A” close to a group of beehives (“C”) where the hornets used to hunt; it was then moved to position “B” at the base of the hill (a vertical slope starts with the dashed line indicated in the picture).

The preliminary check performed in position “A” with a tag mounted on a drone (see Milanesio et al., 2016, for further details on the procedure) already showed a longer detection distance, about 150 m, and a higher number of detections per unit of time as well. As stated while describing both the transponder and the antenna properties, a vertical tag hit by a vertically polarized field always retransmit a signal back to radar, producing a far more continuous trace with respect to the previous setup; in fact, as long as the tag stays in the detection range of the radar and it is not shielded, a returned signal is observed.

The introduction of the telescopic tower helped to have detections beyond closest obstacles, for instance an olive wood located southeast with respect to the radar position “A”; rather, continuous detections were seen about 50 m inside the olive wood, thus indicating that the flight trajectories can be followed in very challenging conditions too. The slightly oscillating movement of the tower itself did not generate additional clutter. Because of the blind range (leakage), detections reported within a 25‐m radius from the system are ignored. Few false detection (mainly cars) were also recorder and then removed, in particular when the radar was in position “B” nearby a quite busy road.

Almost all captured wasps were observed flying right after their release, few of them directed to a blooming medlar tree located in “D”; after a quick recovery, some returned back to the hunting area, some headed out the detection range of the radar along the indicated white arrow (three traces). Few tagged hornets were also recorded coming back to the beehives from that preferential direction (three traces), which roughly corresponds to an existing human trail. A second arrow is also present in Figure 14 indicating another preferential direction used by the insects to escape the radar area (12 traces) and to come back to the hives (three traces), again along an existing trail. This seems to suggest that the flying trajectories are not completely random; on the contrary, they exploit the features of the environment. For instance, very few of the hornets directed northeast from the radar crossed an antihail net, they seemed to prefer to fly around it.

Following this second direction, the radar was then moved to location “B,” about 300 m from the original position. After few minutes of observation, a trace was clearly noted: It corresponded to a wasp crossing the river flowing along the valley, from northeast to southwest; the hornet signal was likely lost due to the presence of a bridge. Unfortunately, we could not locate the nest due to the incoming sunset, but we proved that the system can be moved to locate the position of the hornet nest.

## 4 DISCUSSION

The final goal of this work is to restrain the diffusion of the yellow‐legged Asian hornet in a hilly and woody land such as the Western Liguria; that being so, we upgraded the harmonic radar system firstly described in Milanesio et al. (2016). This entomological radar is able to track the flight of the *V. velutina* specimens, once equipped with proper transponder, in a harsh environment up to 150 m, and to locate their nests.

Harmonic radars for insect tracking have been used in many applications so far (Aniktar et al., 2015; Mazzaro, Martone, Ranney, & Narayanan, 2017). Some were limited in distance (Brazee et al., 2005; Hall & Hadfield, 2009; Mascanzoni & Wallin, 1986; Tsai et al., 2013), others worked very well only in flat environments (Osborne et al., 1999; Riley & Smith, 2002; Riley et al., 1996), but, to the best of our knowledge, none of them could be successfully applied to satisfy our needs. For this reason, starting from what described in Riley and Smith (2002), we developed our own system and fully detailed it in Milanesio et al. (2016). We basically increased the vertical beam of the antennas in order to be able to operate on hilly and rich of obstacles areas, and we adopted a digital processing system of the received signal to increase the detection capability. The first year of testing proved that the radar could work with a maximum range of 125 m, but also pointed out some limitations, in particular about the continuous detectability of the tag. The mounting procedure of the transponder itself appeared to be critical, not only because it stunned the insects for a while, but also because it could considerably influence the probability of detection.

This paper describes the solutions we came up during the second year of testing in order to solve some of those issues, together with some additional options we first followed and eventually abandoned. The upgraded radar has vertically polarized antennas and, therefore, the tag is to be mounted in vertical position on the back of the hornets; this configuration guarantees far more detections per flight as the position of the tag with respect to the radar is no longer critical. Besides, a new mounting procedure has been improved and the time necessary to prepare the insect for the release is reduced to few seconds, with remarkable benefits for the insect itself. The adoption of a movable telescopic tower also allows to quite easily move the radar along the preferential directions of flight of the hornets. Finally, the maximum distance of detection has been increased to 150 m.

In 2017, we expect to test a major upgrade of the system: A new ad hoc transmitting module is being designed from scratch using a new ultra‐wideband (UWB) modulation scheme and a solid‐state power amplifier (SSPA). A new receiving module system with a coherent receiver will be included too. Furthermore, both the TX and the RX antennas will be upgraded by increasing the number of radiating elements and, therefore, the correspondent antenna gains.

## CONFLICT OF INTEREST

None declared.
